# Cannabidiol Isolated From *Cannabis sativa* L. Protects Intestinal Barrier From *In Vitro* Inflammation and Oxidative Stress

**DOI:** 10.3389/fphar.2021.641210

**Published:** 2021-04-28

**Authors:** Veronica Cocetta, Paolo Governa, Vittoria Borgonetti, Mattia Tinazzi, Gregorio Peron, Daniela Catanzaro, Massimiliano Berretta, Marco Biagi, Fabrizio Manetti, Stefano Dall’Acqua, Monica Montopoli

**Affiliations:** ^1^ Department of Pharmaceutical Sciences, University of Padova, Padova, Italy; ^2^ Department of Biotechnology, Chemistry and Pharmacy Department of Excellence 2018-2022, University of Siena, Siena, Italy; ^3^ Department of Neuroscience, Psychology, Drug Research and Child Health (NEUROFARBA), Section of Pharmacology, University of Florence, Florence, Italy; ^4^ Department of Clinical and Experimental Medicine, University of Messina, Messina, Italy; ^5^ Department of Physical Sciences, Earth and Environment, University of Siena, Siena, Italy; ^6^ Veneto Institute of Molecular Medicine, VIMM, Padova, Italy

**Keywords:** intestinal barrier dysfunction, Cannabis sativa, cannabidiol, intestinal inflammation, transepithelial electrical resistance, intestinal permeability

## Abstract

The relevance and incidence of intestinal bowel diseases (IBD) have been increasing over the last 50 years and the current therapies are characterized by severe side effects, making essential the development of new strategies that combine efficacy and safety in the management of human IBD. Herbal products are highly considered in research aimed at discovering new approaches for IBD therapy and, among others, *Cannabis sativa* L. has been traditionally used for centuries as an analgesic and anti-inflammatory remedy also in different gastrointestinal disorders. This study aims to investigate the effects of different *C. sativa* isolated compounds in an *in vitro* model of intestinal epithelium. The ability of treatments to modulate markers of intestinal dysfunctions was tested on Caco-2 intestinal cell monolayers. Our results, obtained by evaluation of ROS production, TEER and paracellular permeability measurements and tight junctions evaluation show Cannabidiol as the most promising compound against intestinal inflammatory condition. Cannabidiol is able to inhibit ROS production and restore epithelial permeability during inflammatory and oxidative stress conditions, suggesting its possible application as adjuvant in IBD management.

**GRAPHICAL ABSTRACT:**
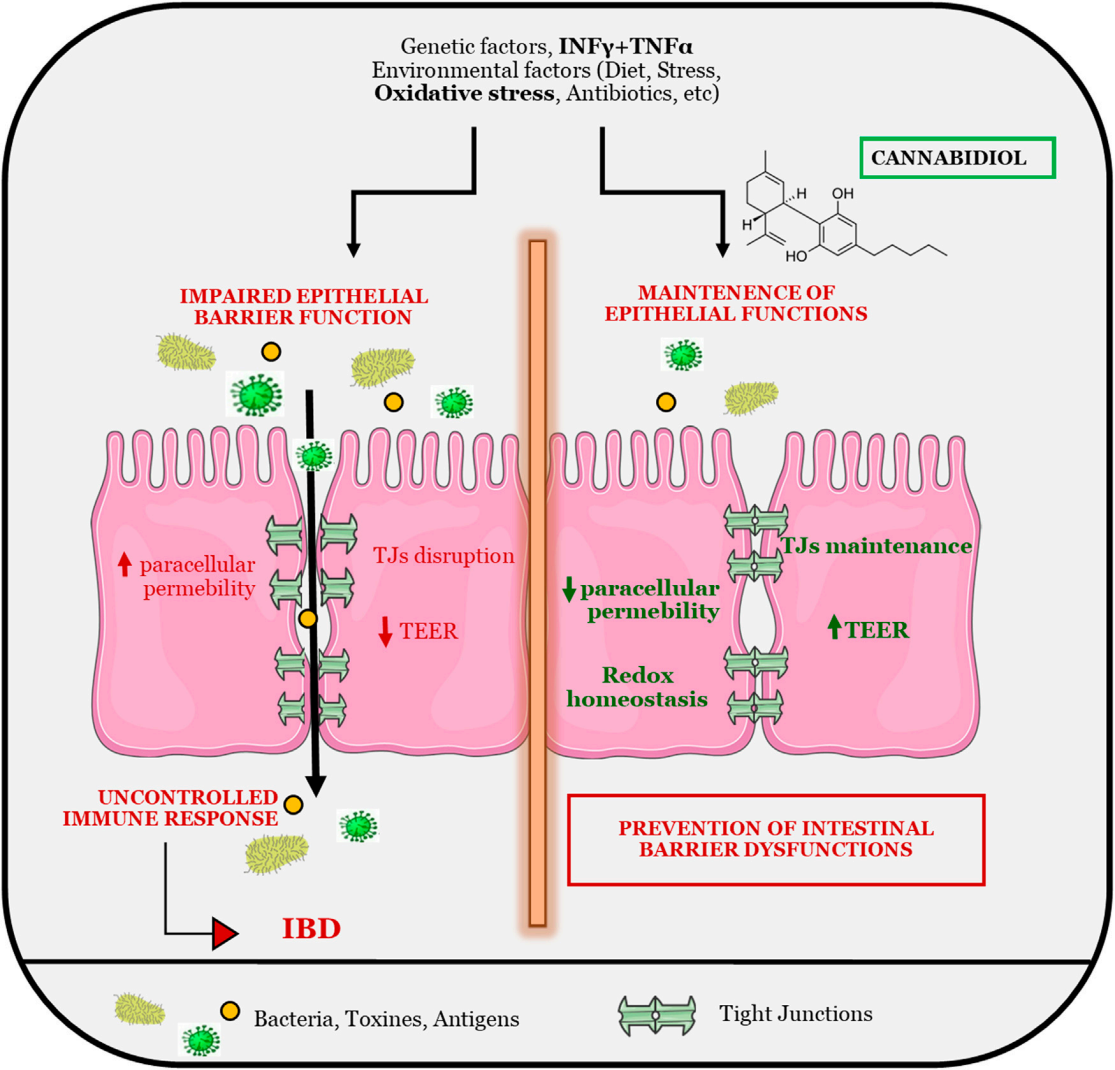


## Introduction

The gastrointestinal epithelium forms the body’s larger interface between the external environment providing a functional epithelial barrier that regulates the bi-directional flow of water, ions and macromolecules between the lumen and the host. The barrier selectively allows absorption of water and nutrients, while limiting the permeation of toxins and antigens (Schmitz et al., 1999; Ahmad et al., 2017). A breach in the mucosal barrier incites mucosal inflammation leading to a wide array of non-intestinal and intestinal disorders including clinically diagnosed inflammatory bowel disease (IBD). The selective barrier is regulated by both transcellular and paracellular transport mechanisms but is the paracellular pathway that has received the most attention for its role in regulation of mucosal permeability (D’Incà et al., 1999; Su et al., 2009) and initial clinical observation indicate that intestinal epithelium of IBD patients is more permeable to paracellular-permeable traces molecules (Hering, et al., 2012). These findings, also confirmed in mouse models, support a correlation between the mucosal leakiness and mucosal inflammatory conditions, suggesting that deregulation of intestinal barrier functions by dietary, microbial and immunological factors might precede the clinical IBD manifestation.

The selective barrier is provided by precise physical approximation between enterocytes, which is reinforced by tight junctions (TJs), the main responsible for paracellular permeability regulating apical cell-cell adhesion. Moreover, the intestinal barrier is furnished with immune and non-immune molecules, including mucosal immunoglobulins that contributed to the maintenance of gut homeostasis (Odenwald and Turner 2017).

The prevalence of IBD is around one in 1,000 people in Europe, with increasing incidence in westernized and industrialized countries (Geremia et al., 2014; Ng et al., 2018). The two most common types of IBD are Crohn’s disease (CD) and ulcerative colitis (UC) which, although rarely fatal, can greatly impair the quality of life, causing abdominal pain, vomiting, diarrhea, weight loss and increased risk of colorectal cancer (May et al., 2011; Choi et al., 2017).

The current therapeutic choices for the management of IBD are focused on the symptomatology to induce and maintain the suppression of aberrant immune response. The pharmacological treatment includes corticosteroids, immunosuppressant agents and biological therapies with anti-tumor necrosis factor-alpha (TNFα) antibody as the mainstream treatment for down-regulating immune responses and inflammatory cascades (Hemperly et al., 2018). Nevertheless, the treatment is accompanied with adverse and side effects that strongly impair patient’s quality of life. The etiology of these diseases is uncertain and not yet full elucidated. The current knowledge links the onset of IBD with a complex combination of causes such as multiple genetic variations, environmental changes, infectious and psychological factors, that lead to alterations in intestinal epithelial barrier and composition of the intestinal microbiota, breaking out in over activity of the intestinal mucosal immune response (De Mattos et al., 2015).

It is therefore of particular interest to identify and develop new therapies that combine efficacy and safety for the management of these inflammatory chronic pathologies.

Herbal products are among the most relevant types of complementary and alternative medicine used for the treatment of IBD (Holleran et al., 2020; Musumeci et al., 2020; Hossen et al., 2020; Algieri et al., 2015). *Cannabis sativa* L. has been used for many centuries to treat a variety of gastrointestinal conditions such as inflammation, infections, pain, disorders of motility and vomiting (Hasenoehrl et al., 2016; Coutts and Izzo 2004; Sanger 2007; Izzo and Camilleri 2008; Pellati et al., 2018; Borgonetti et al., 2019). Numerous studies had identified the presence of a functional endocannabinoid system in the gut of several mammals including humans; it has also been demonstrated that the tone of endocannabinoid system is increased during inflammation because of either increased expression of cannabinoid receptors and/or upregulation of endocannabinoid levels (di Marzo and Izzo, 2006; Guida et al., 2020; Jansma et al., 2020). In particular, CB1 receptor expression has been identified in the enteric nervous system and it can give reason for the cannabinoids’ activity in the gastrointestinal tract (Coutts et al., 2002; Mehrpouya-Bahrami et al., 2017).

Nevertheless, the therapeutic utility of *C. Sativa* is limited by the occurrence of psychoactive effects, prevalently due to the presence of Δ^9^-tetrahydrocannabinol (THC), which activates CB1 receptors in brain (Di Marzo, 2008; National Academies of Sciences, Engineering, and Medicine, 2017). On the other hand, other *C. sativa* constituents, such as cannabidiol (CBD), are free from this kind of central effects, having low affinity for both CB1 and CB2 receptors (Borrelli et al., 2009; Vučković et al., 2018).

The main aim of this work is therefore to investigate the potentiality of *C. sativa* extracts and its main cannabinoids in the control of intestinal barrier permeability alterations and gut inflammation, furnishing further details about the potential use of *Cannabis sativa* as coadjuvant in IBD management.

## Materials and Methods

### Extraction and Quantification of Cannabinoids

CBD, THC, cannabidiolic acid (CBDA) and tetrahydrocannabinolic acid (THCA) analytical standards were purchased from Sigma Aldrich. *Cannabis sativa* supercritical carbon dioxide (scCO_2_) extracts were provided by a local producer. The scCO_2_ extract was obtained from the aerial parts of *Cannabis sativa* L. cultivated in the North-Eastern region of Italy (Veneto region), using scCO_2_ at 280 bar and 42°C. The decarboxylated extract was obtained by heating the scCO_2_ extract at 150°C for 5 h and controlling the modification of the extract composition by TLC and HPLC.

Stock standard solutions were prepared in methanol at concentrations of 1 mg/mL and stored in the dark at −20°C; the working standard solutions of CBD and THC were diluted in methanol with a concentration of 100, 50, 10 and 1 μg/mL to prepare the calibration curves.

The total extract was analyzed by HPLC-DAD (high-pressure liquid chromatography coupled with diode array detector) to quantify the content in active compounds. The sample was prepared dissolving 40 mg of the extract in 25 mL of ethanol with an ultrasonic treatment for 20 min. After centrifugation (15 min, 13,000 rpm) the supernatant was transferred in 1.5 mL vials for the analysis. The main cannabinoids were identified using reference literature (De Backer et al., 2009), and identification was confirmed by co-injection with reference standards, when possible.

For the HPLC analysis, an Agilent 1260 binary pump equipped with a 1260 auto-sampler, column oven and DAD 1260 series detector was used. Separation was achieved using an Agilent Eclipse XDB C-18 (4.6 × 250 mm, 5 µm) column as stationary phase. The binary gradient of elution using aqueous formic acid 0.1% (A) and acetonitrile (B) was as follows: from 65 to 100% of B in 30 min, then to 65% of B in 1 min and isocratic up to 36 min. The flow rate was set at 1 mL/min and injection volume was 10 μl.

### Isolation of Cannabinoids by Semi-Preparative HPLC and Characterization

For preparative HPLC, the sample was prepared dissolving the extract in ethanol with a final concentration of 10 mg/mL, using an ultrasonic bath for 20 min.

The preparative HPLC system consisted of a Varian 920 HPLC with quaternary pump equipped with UV-Vis detector. The chromatographic separation was performed on an Agilent Zorbax SB C-18 column (21.2 × 150 mm, 5 µm). The mobile phase was delivered at a flow rate of 5 mL/min. The chromatographic run was performed with a binary, linear A/B gradient (solvent A: 0.1% formic acid in water; solvent B: acetonitrile). The program was as follows: 0 min, 65% B; 30 min, 100% B; 34 min, 100% B; 35 min, 65% B, and isocratic up to 38 min. The injection volume was 30 μl. Each peak was collected, and the obtained fractions were evaporated to dryness under vacuum on a rotary evaporator at 60°C. The dry residues were re-dissolved in deuterated chloroform for characterization by NMR analysis.

^1^HNMR, COSY, TOCSY, HSQC, and HMBC spectra were recorded on a Bruker Avance III spectrometer (400 MHz), using standard pulse sequences. NMR analyses were performed on the whole scCO_2_ extract and on all the purified fractions. 2D spectra were processed with Topspin 4.0.6 (Bruker) using zero filling to 1024 in F1 dimension, squared sine-bell apodization in both dimensions, prior to Fourier transformations.

### Intestinal Cell Monolayer Preparation and Treatment

Caco-2 cells, obtained from ATCC, were grown in high glucose Dulbecco’s modified Eagle’s media (DMEM) supplemented with 10% FBS, 1% l-glutamine and 1% penicillin/streptomycin. Cells were maintained at 37°C under a humidified atmosphere of 5% CO_2_ in air. Experimental inflammatory condition in Caco-2 cell monolayers was induced by exposure for different times according to the assays, to 10 ng/mL recombinant human IFN-γ (Sigma-Aldrich) for 3 h and then 10 ng/mL TNF-α (Sigma-Aldrich) or to 500 μM H_2_O_2_, as previously described (Catanzaro et al., 2015). A 24 h pre-treatment with CBD, CBDA, THC and THCA (0.01–10 μg/mL) was applied before the stimulation. Reagents for cell cultures were from Cambrex-Lonza (Basel, Switzerland) and FBS from Gibco, Invitrogen (Carlsbad, CA).

### Cell Viability Assay

Cell viability was determined by the 3-(4,5-dimethyl-thiazole-2-yl)-2,5-diphenyltetrazolium bromide (MTT, Sigma-Aldrich) assay, as previously described (Montopoli et al., 2011). Briefly, Caco-2 cells (5 × 10^3^) were seeded in 96-multiwells culture plates and treated with CBD, CBDA, THC and THCA (0.01–10 μg/mL) for 24-48-72 h. Cells were then washed, and fresh medium was added. MTT (5 mg/mL) was added to each well and incubated for 4 h at 37°C. Cells were lyzed with acidic isopropanol and the formazan absorbance was measured at 570 nm, using a Multilabel Plate Reader VICTOR™ X3 (Perkin Elmer).

### ROS Fluorescence Assay

ROS were quantified using 2′,7′-dichlorofluorescein-diacetate (H_2_-DCF-DA, Sigma-Aldrich), as previously described (Catanzaro et al., 2015). The H_2_−DCF-DA is converted to the fluorescent 2′,7′-dichlorofluorescein (DCF) upon cleavage of acetate groups by intracellular esterase and oxidation. Briefly, Caco-2 cells (5 × 10^3^) were seeded into 96-well plates and allowed to adhere overnight. 24 h treatment with CBD, CBDA, THC and THCA (0.01–10 μg/mL) was applied, followed by addition of 50 μM H_2_-DCF-DA, incubation for 30 min at 37°C and wash with phosphate-buffered saline (PBS). DCF fluorescence intensity was measured at excitation 485 nm—emission 535 nm, using a Multilabel Plate Reader VICTOR™X3 (Wallac Instruments, Turku, Finland) in absence or presence of 500 μM H_2_O_2_. Fold increase in ROS production was calculated using the equation: $\left(F_{treatment}-F_{blank}/F_{control}-F_{blank}\right)$, where *F* is the fluorescence reading.

### Transepithelial Electrical Resistance (TEER) Assay

Caco-2 cells (15 × 10^4^) were seeded on Transwell™ polyester membrane cell culture inserts (transparent PET membrane: 1.0 cm^2^ growth surface area, 0.4 μm pore size; BD Falcon™) in 24-well plates and incubated with DMEM at 37°C in a humidified atmosphere and 5% CO_2_. Culture media was replaced every two days until confluent monolayer was obtained. The integrity of the cell monolayers was monitored by measuring the transepithelial electric resistance (TEER) from day 14th to day 21st after seeding. A 24 h pre-treatment was done adding CBD or THC (0.01–0.1 ug/mL) in the apical chamber. The TEER assay was performed in Hanks’ Balanced Salt solution (HBSS, Cambrex Lonza) with 10 mM Hepes and 10 mM d-glucose (pH = 7.4), after an equilibration period at RT (Liu et al., 2010). Treatments were added to the apical chamber and inflammatory stimuli to the basal chamber. Millicell^®^ ERS meter (Millipore Corporation) connected to a pair of chopstick electrodes were inserted in the donor and receiver chambers and the 24 h-time courses of TEER variation was recorded (1-3-6-21-24 h). TEER was expressed as percentage of resistance, normalized to initial value (Governa et al., 2018).

### Paracellular Permeability Assay

Fluorescein isothiocyanate flux across Caco-2 cell monolayers was used as measure of the paracellular permeability. After recording of the 24 h TEER variation, the apical medium was replaced with a solution of fluorescein isothiocyanate 0.1 mM in HBSS. After 30 min incubation at 37°C, 200 μl of medium were taken from the basal chamber and the amount of fluorescein permeated was measured using a Multilabel Plate Reader VICTOR X3 (PerkinElmer) at excitation 480 nm—emission 530 nm.

### Immunofluorescence Microscopy

15 × 10^5^ cells were seeded on glass coverslips precoated with gelatin (Sigma-Aldrich, St Louis, MO) in 24-well plates, allowed to attach and reach the confluence for 5 days. Cells were then pretreated for 24 h with CBD and THC (0.1–1 μg/mL) following by addition of inflammatory stimulus (IFNγ 10 ng/mL for 3 h and TNFα 10 ng/mL for 21 h). At the end of treatment, cells were washed, fixed with 4% formaldehyde, permeabilized with 0.1% Triton X-100 in PBS and stained for 1 h at 37°C with mouse monoclonal anti-occludin antibody (Invitrogen Life Technologies), rabbit anti-ZO1 antibody (Invitrogen Life Technologies). After PBS wash, cells were incubated with secondary antibodies/fluorescein isothiocyanate (Alexa Fluor 488 anti-mouse or Cy5 anti-rabbit immunoglobulin G, Molecular Probes, Invitrogen Life Technologies) for 1 h at 37°C. After washing, cells were incubated for 20 min with Hoechst (1:10,000) at RT. Th coverslips were then mounted on glass slides by using Mowiol 40–88 (Sigma-Aldrich) and images were acquired through confocal microscope LSM 800, magnification ×60, software ZN 2.1 blue Edition (Carl Zeiss, Jenza).

### Statistical Analysis

The statistical analysis was performed using GraphPad Prism version 3 for Windows (GraphPad Software, San Diego, CA). Results are presented as mean ± SEM. Standard ANOVA procedures were performed for all the cell viability; the unpaired Student’s *t*-test was used to compare ROS values, TEER values and paracellular permeability; *p* values < 0.05 were considered statistically significant.

## Results

### Characterization and Isolation of Cannabinoids From *C. sativa* scCO_2_ Extract

**FIGURE 1:**
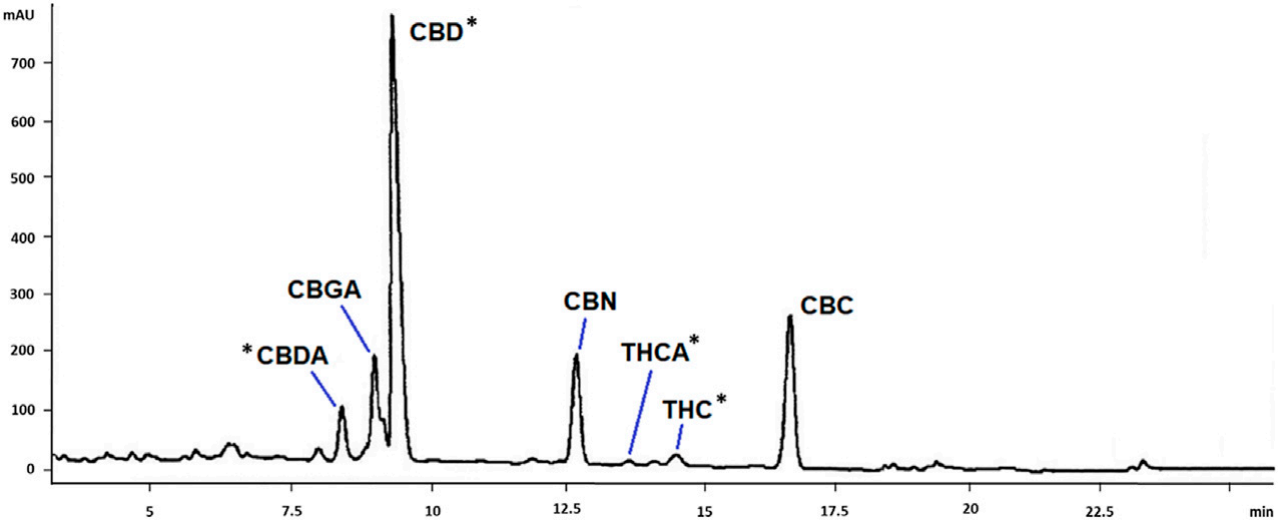
HPLC-DAD chromatogram of *C. sativa* scCO_2_ extract. Tentative identification of the main cannabinoids are reported in the Figure. CBDA, cannabidiolic acid; CBGA, cannabigerolic acid; CBD, cannabidiol; CBN, cannabinol; THCA, tetrahydrocannabinolic acid; THC, tetrahydrocannabinol; CBC, cannabichromene. *: identification was confirmed by co-injection with reference standards.

**FIGURE 2:**
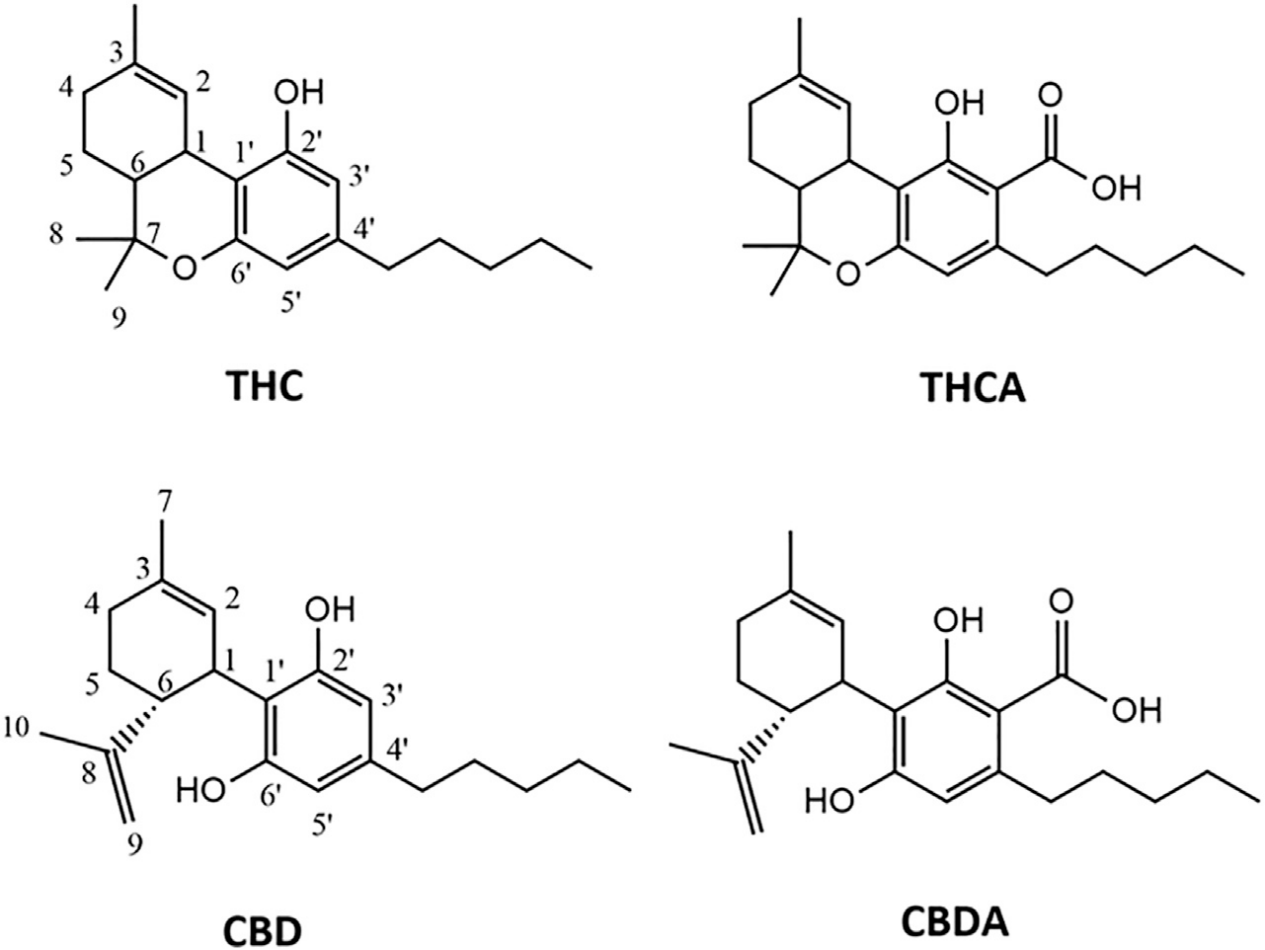
Chemical structures of the cannabinoids isolated from *C. sativa* scCO_2_ extract. THC, tetrahydrocannabinol; THCA, tetrahydrocannabinolic acid; CBD, cannabidiol; CBDA, cannabidiolic acid.

Cannabinoids in scCO_2_ extract were quantified by HPLC-DAD. A representative chromatogram is reported in Figure 1. Compound identification was achieved by comparison of retention time and UV-Vis spectra with previously published literature (De Backer et al., 2009). The main cannabinoids identified by comparison with reference standards were CBD, CBDA, THC and THCA (Figure 2). These latter were the only quantified based on calibration curves built upon injection of reference standards, and they were further considered for the biological studies. The amounts of these compounds in the scCO_2_ extract are reported in Table 1.

**TABLE 1:** Amounts of the main cannabinoids in *C. sativa* scCO_2_ extract, expressed as percentages of whole extract.

	CBD (% w/w)	CBDA (% w/w)	THC (% w/w)	THCA (% w/w)
Total scCO_2_ extract	17.54 ± 0.03	8.41 ± 0.02	0.87 ± 0.01	0.11 ± 0.01

Pure CBD, CBDA, THC and THCA were isolated from the scCO_2_ extract using a semi-preparative HPLC method. The chromatographic gradient was maintained the same utilized for the characterization of cannabinoids in the scCO_2_ extract, to reproduce the elution of the peaks associated to each cannabinoid. The four isolated compounds, namely CBD, CBDA, THC and THCA, were characterized by ^1^HNMR, comparing the spectra with previously published literature (Choi et al., 2004).

The first compound, CNP-1, was assigned to CBDA. Characteristic signals in the NMR spectrum (reported in Table 2) are a singlet at δ 11.97 assignable to a carboxyl acid proton, a singlet at δ 5.56, two multiplets at δ 4.55 and 4.40 and two singlets integrating for three protons at δ 1.80 and δ 1.72. Finally, a triplet integrating for three protons at δ 0.90 was detected. COSY and HSQC analyses indicated that CNP-1 is characterized by six CH_2_ groups, one sp2 CH_2_, two aliphatic CH and two sp2 CH. The compound contains a five-atom aliphatic chain characterized by a triplet at δ 0.90 ascribable to the terminal CH_3_, and by signals at δ 1.35, δ 1.57 and δ 2.81 ascribable to intra-chain CH_2_ groups. The latter signal can be assigned to a more de-shielded CH_2_, bonded to the aromatic ring of the core structure, as confirmed by the HMBC correlations between the signal at δ 2.81 (H-1″) with those at δ 146.8 (C-4′), δ 112.0 (C-5′) and δ 103.5 (C-3′). Other significant correlations could be observed between the singlet at δ 6.26 (H-5′) and the signals at δ 114.5 (C-1′) and δ 175.0 (COOH). Diagnostic HMBC correlations of the core cyclohexane are those between the signal at δ 1.80 (CH_3_ in position 7) and those at δ 123.7 (C-2), δ 30.8 (C-4) and δ 139.5 (C-3). The signal at δ 5.56 assignable to the H-2 proton correlates with those at δ 31.7 and δ 37.4, ascribable to C-5 and C-1 carbons, respectively. Finally, other correlations are observed between the sp2 CH_2_ of the cyclohexane ring (H-9, δ 4.40 and 4.55) and C-5, C-6 and C-10 carbons (δ 31.7, δ 46.5 and δ 18.4, respectively).

**TABLE 2:** Characteristic ^1^HNMR and ^13^CNMR signals for cannabidiolic acid (CBDA) and cannabidiol (CBD).

	CBDA		CBD	
Position	δ ^1^HNMR (multiplicity)	δ^13^CNMR	δ ^1^HNMR (multiplicity)	δ^13^CNMR
1	3.91 (m)	37.4	4.09 (m)	37.4
2	5.56 (s)	123.7	5.55 (s)	123.8
3	-	139.5	-	139.4
4	2.19 (m)	30.8	2.19 (m)	29.3
5	1.87 (m)	31.7	1.89 (m)	31.7
6	2.39 (m)	46.5	2.39 (m)	46.0
7	1.80 (s)	23.5	1.81 (s)	23.5
8	-	148.8	-	148.8
9	4.55 and 4.40 (m)	110.5	4.64 and 4.54 (m)	110.0
10	1.72 (s)	18.4	1.67 (s)	18.4
1′	-	114.5	-	114.5
3′	-	103.5	6.26 (s)	107.5
4′	-	146.8	-	142.8
5′	6.26 (s)	112.0	6.27 (s)	109.0
1″	2.81 (m)	37.8	2.39 (m)	37.8
2″	1.57 (m)	32.8	1.57 (m)	31.8
3″	1.35 (m)	33.0	1.30 (m)	32.9
5″	0.90 (t)	14.1	0.90 (t)	14.1
COOH	11.97 (s)	175.0	-	-

The second compound, CNP-2, showed similar NMR signals to those of CNP-1 (Table 2). The differences were observable for signals attributable to H-9, namely at δ 4.54 and 4.64, and for H-1, assigned to a multiplet at δ 4.09. Furthermore, a signal integrating for one proton at δ 6.26 was assigned to H-3′. HSQC-DEPT and COSY experiments indicated the presence of three methylene groups (among which, two tertiary), seven CH_2_ (among which, one sp2), and five CH, among which two sp2. Diagnostic HMBC correlations could be observed between the signal at δ 1.81 (attributable to the CH_3_ in position 7) and those at δ 139.4, δ 123.8, and δ 29.3, assignable respectively to the carbons C-5, C-8 and C-4. Overall, these data support the assignment of CBD for CNP-2 fraction (Choi et al., 2004).

The NMR spectra of compound CNP-3 allowed to assign it to THCA. Distinctive signals are reported in Table 3. Characteristic singlets integrating for 3H were observed at δ 1.44 and δ 1.11 (Choi et al., 2004), and they were assigned to the protons in positions 8 and 9. Other characteristic signals were the singlet at δ 6.26 integrating for 1H, the singlet at δ 1.68 integrating for 3H, and the multiplet at δ 2.17 integrating for 2H. These were assigned respectively to H-5′, to the methyl group in position 7, and to H-4. Another characteristic signal is the singlet at δ 12.20, corresponding to the proton of the hydroxyl group in position 2′, which allows to differentiate the compound from its decarboxylated form (Choi et al., 2004). HMBC allowed to assign the carbon signals at δ 109.7, δ 164.4, δ 102.1, δ 146.8 and δ 112.3 to C-1′, C-2′, C-4′, C-5′, and C-6′, respectively. Comparison with the spectrum of CNP-4 (THC) allowed to highlight characteristic differences between the two compounds, as already reported (Choi et al., 2004).

**TABLE 3:** Characteristic ^1^HNMR and ^13^CNMR signals for tetrahydrocannabinolic acid (THCA) and Δ^9^-tetrahydrocannabinol (THC).

	THCA		THC	
Position	δ ^1^HNMR (multiplicity)	δ^13^CNMR	δ ^1^HNMR (multiplicity)	δ^13^CNMR
1	3.23 (m)	33.5	3.20 (m)	33.6
2	6.39 (s)	123.2	6.31 (m)	123.6
3	-	133.8	-	133.9
4	2.17 (m)	31.2	2.16 (m)	31.0
5	1.92 (m)	25.0	1.89 (m)	25.0
6	1.67 (m)	45.6	1.69 (m)	45.7
7	1.68 (s)	78.8	1.69 (s)	77.6
8	1.44 (s)	27.6	1.42 (s)	29.1
9	1.11 (s)	19.2	1.10 (s)	18.9
1′	-	109.7	-	110.8
2′	-	164.4	-	155.7
3′	-	102.1	6.15 (s)	107.5
4′	-	146.8	-	142.8
5′	6.26 (s)	112.3	6.28 (s)	109.0
6′	-	159.8	-	154.0
1″	2.94 (m)	36.5	2.39 (m)	35.3
2″	1.57 (m)	31.3	1.32 (m)	29.5
3″	1.35 (m)	32.0	1.27 (m)	30.9
4″	1.35 (m)	22.0	1.28 (m)	21.8
5″	0.90 (t)	14.1	0.88 (t)	15.1
2′-OH	12.19 (s)	-	4.90 (s)	-
COOH	11.97 (s)	176.2	-	-

Finally, the spectrum of compound CNP-4 showed the characteristic signals of THC at δ 6.31, δ 6.28 and δ 6.15 integrating one proton each, and three singlets at δ 1.69, δ 1.42 and δ 1.10 integrating for three protons (Table 3). The compound is characterized by a 5-atoms aliphatic chain, as indicated by the triplet at δ 0.88 assignable to the terminal CH_3_ (H-5″), and the signals at δ 1.27 and δ 1.32 attributable to the intra-chain CH_2_ moieties. HSQC-DEPT analysis allowed to assign the signal at δ 0.88 to the proton H-5″ bonded to the carbon at δ 15.07, confirming its assignation. On the same way, H signals at δ 1.10 and δ 1.69 were associated to H-9 and H-3 bonded to carbons at δ 18.94 and δ 77.6, respectively. HMBC allowed to correlate the signal at δ 0.88 with the signals at δ 21.8 and δ 30.92 corresponding to C-4″ and C-3″, respectively, and the one at δ 1.27 with the carbon signal at δ 29.50 (C-2″), confirming the presence of the aliphatic chain. Diagnostic HMBC correlations for methyl groups were also observed: H-9 (δ 1.10) is correlated to C-8 (δ 29.08), C-6 (δ 45.7), and to a signal at δ 77.6 attributable to C-7. The proton in 8 (δ 1.42) correlates with C-6 (δ 45.7) and C-7 (δ 77.6), while the protons of the CH_3_ in position 7 (δ 1.69) correlate with C-4 (δ 31.02), C-2 (δ 123.6) and C-3 (δ 133.9).

The NMR spectra and HPLC run also confirmed the purity of isolated fractions.

### Effect of *C. sativa* Isolated Cannabinoids on Caco-2 Cells Viability

**FIGURE 3:**
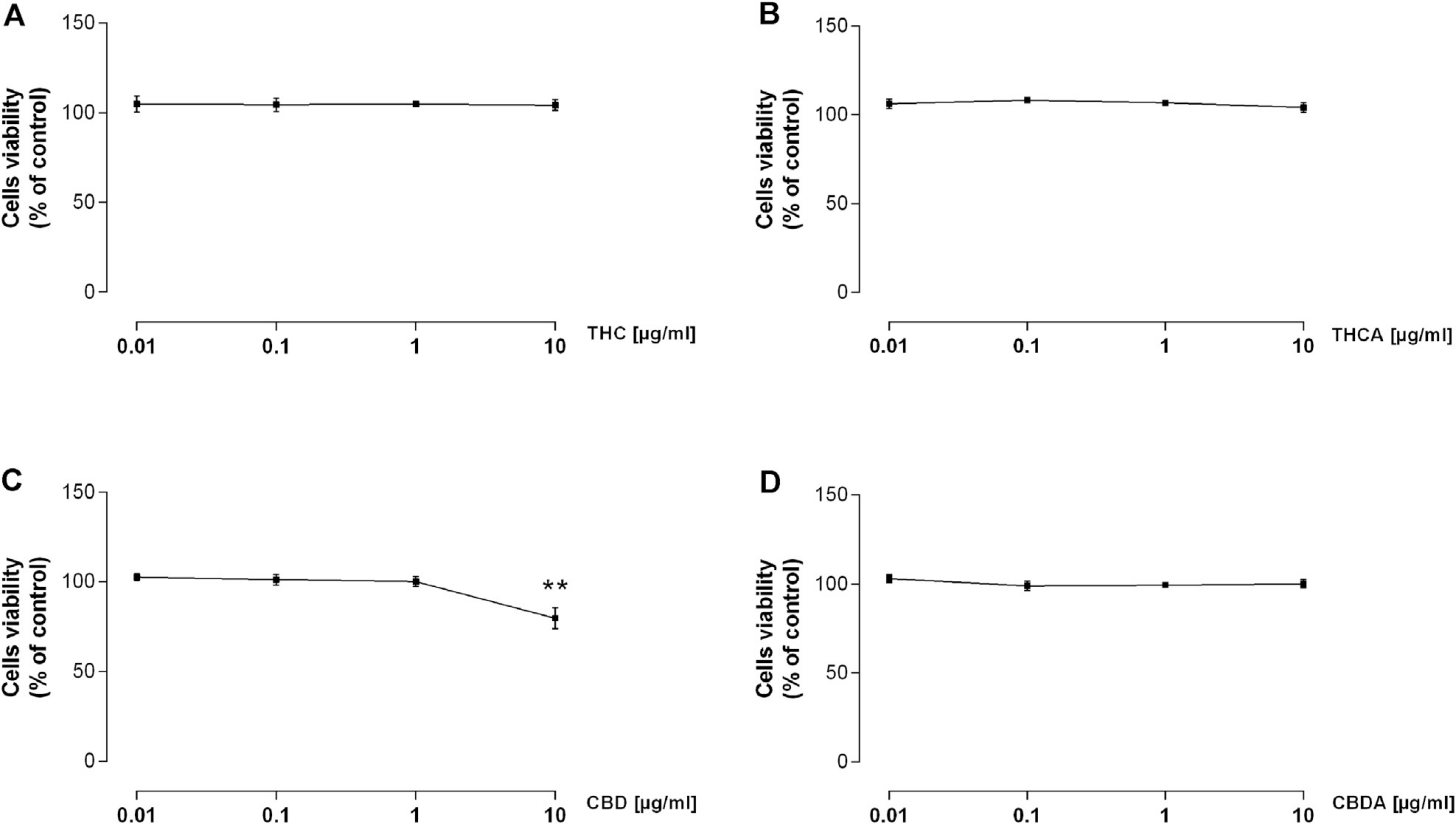
Effect of a 24 h treatment with THC **(A)**, THCA **(B)**, CBD **(C)**, and CBDA **(D)** 0.01-0.1-1-10 μg/mL on Caco-2 cells viability. Results are the mean ± SEM of *n* = 3 experiments and are expressed as percentage of absorbance of treated cells related to control. **p < 0.01 treatment vs. control.

As reported in Figure 3, none of the tested compounds affected the viability of the intestinal cells, with exception for the highest concentrations of CBD, which significantly reduced cell viability by approximately 20% (Figure 3C; Supplementary Figure S1).

### THC and CBD Reduced ROS Production in Basal and Oxidative Condition

**FIGURE 4:**
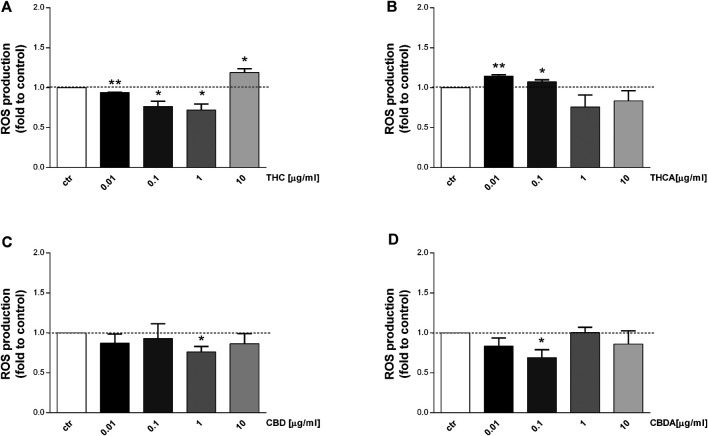
Effect of a 24 h treatment with THC **(A)**, THCA **(B)** CBD **(C)**, CBDA **(D)** (0.01-0.1-1-10 μg/mL), on Caco-2 ROS production in basal condition. Data are expressed as mean ± SEM of fluorescence intensity (FI) of treated cells related to control. *n* = 3 experiments. *p < 0.05, **p < 0.01, ***p < 0.001 treatment vs. control, Standard ANOVA procedures.

**FIGURE 5:**
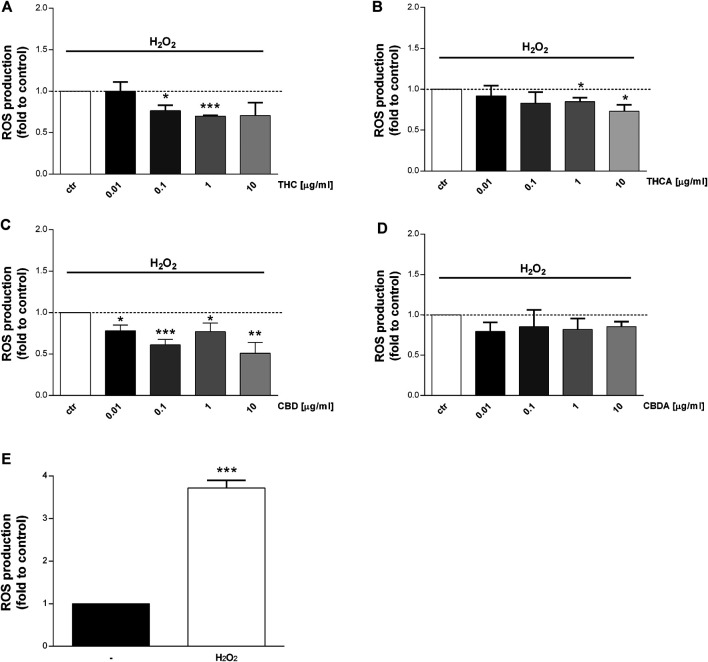
Effect of a 24 h treatment with THC **(A)**, THCA **(B)** CBD **(C)**, CBDA **(D)** (0.01-0.1-1-10 μg/mL), on Caco-2 ROS production after oxidative simulation with H_2_O_2_ **(E)**. Data are expressed as mean ± SEM of fluorescence intensity (FI) of treated cells related to control. *n* = 4 experiments. *p < 0.05, **p < 0.01, ***p < 0.001 treatment vs. control, unpaired Student’s *t*-test.

Consistent increase of reactive oxygen and nitrogen species (ROS-NOS) production and increased epithelial permeability are among the factors implicated in IBD pathogenesis (Moura et al., 2015; de Souza and Fiocchi 2016). The effect of *Cannabis* extracts on ROS production is depicted in Figures 4, 5. In basal condition, THC decreased ROS levels by 25 and 30% at 0.1 and 1 μg/mL, respectively (Figure 4A), though no effect was observed for THCA (Figure 4B). CBD 1 μg/mL was able to reduce ROS levels by 25% compared to control (Figure 4C), while CBDA resulted more potent, reducing ROS production by 30% at 0.1 μg/mL (Figure 4D).

Interestingly, an increase in ROS levels was observed following treatment with THC at 10 μg/mL, and with THCA at 0.01 μg/mL (Figures 4A,B). The effect of the compounds has been tested also in oxidative stress conditions (stimulation with H_2_O_2_) which induce a significant increase in ROS level (Figure 5E). CBDA was not able to modulate the increase in ROS production induced by the exposure to H_2_O_2_ (Figure 5D). Nevertheless, CBD significantly counteracted the oxidative stimulus, at each of the tested concentration (Figure 5C).

THC resulted to be more potent than THCA, reducing H_2_O_2_-induced ROS production by 25 and 30% at 0.1 and 1 μg/mL, respectively (Figures 5A,B).

### CBD, but not THC, Protects Caco-2 Monolayer Integrity from Oxidative and Inflammatory Stimulation

TEER and paracellular permeability were evaluated in Caco-2 monolayer in basal condition and after exposure to oxidative or inflammatory stimulus (H_2_O_2_ or INFγ+TNFα). Only the treatments which resulted in a reduction of ROS production in both basal and oxidative condition (i.e., THC and CBD at two different concentration, 0.1 and 1 μg/mL) were applied.

**FIGURE 6:**
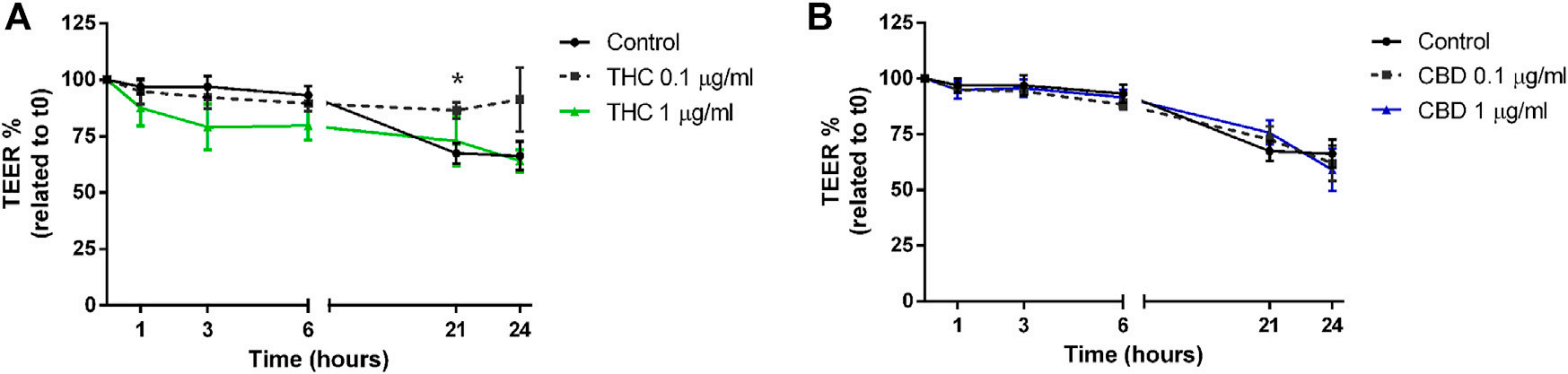
Effects of THC **(A)** and CBD **(B)** 0.1–1 μg/mL on transepithelial electrical resistance in Caco-2 cells monolayer. Data are expressed as mean ± SEM percentage of baseline TEER value of *n* = 3–5 experiments, *p < 0.05 treatment vs. control, unpaired Student’s *t*-test.

As reported in Figure 6A, THC induces a tendency to reduction of TEER levels compared to control, at every time point, except for the 0.1 μg/mL concentration, that after 21 and 24 h seems to increase TEER values. CBD, instead, maintains the TEER levels comparable to control for all the time course of measurements (Figure 6B). This trend may suggest a protective role of both THC and CBD on intestinal epithelium, in the long-term use.

**FIGURE 7:**
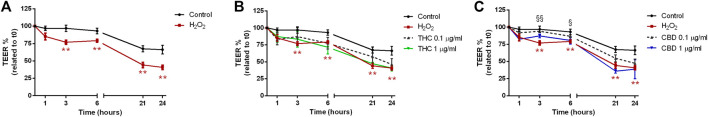
Effects of THC **(B)** and CBD **(C)** 0.1–1 μg/mL on transepithelial electrical resistance in Caco-2 cells monolayer stimulated with H_2_O_2_ **(A)**. Data are expressed as mean ± SEM percentage of baseline TEER value of *n* = 3–5 experiments. **p < 0.01 oxidative stimulus vs. control; §*p <* 0.05; §§*p <* 0.01 treatment vs. oxidative stimulus, unpaired Student’s *t*-test.

The exposure to H_2_O_2_ (Figure 7) leads to a significant reduction of TEER after 3-6-21 and 24 h of treatment (Figure 7A). While THC was not able to counteract the H_2_O_2_-induced oxidative effect (Figure 7B), CBD at 0.1 μg/mL resulted to be effective in ameliorating the TEER decrease induced by the oxidative stimulus for all the time course, with significant results after 3 and 6 h of treatment (Figure 7C).

**FIGURE 8:**
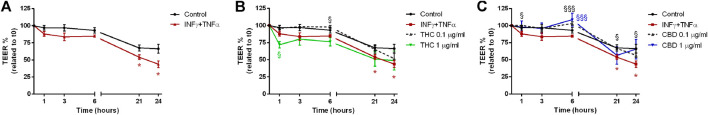
Effects of THC **(B)** and CBD **(C)** 0.1–1 μg/mL on transepithelial electrical resistance in Caco-2 cells monolayer stimulated with INFγ+TNFα **(A)**. Data are expressed as mean ± SEM percentage of baseline TEER value of *n* = 3–5 experiments. *p < 0.05 inflammatory stimulus vs. control; §*p <* 0.05; §§§*p <* 0.001 treatment vs. oxidative stimulus, unpaired Student’s *t*-test.

When Caco-2 monolayer was exposed to the inflammatory stimulus induced by INFγ+TNFα (Figure 8), a reduction of membrane integrity was recorded at each time point, significantly at 21 and 24 h (Figure 8A). THC had a good protective effect only at 0.1 μg/mL (Figure 8B). On the contrary, CBD was effective at both 0.1 and 1 μg/mL maintaining the TEER values comparable with the control (Figure 8C).

**FIGURE 9:**
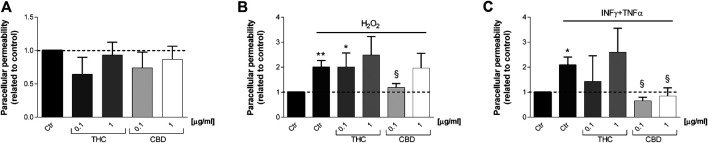
Effects of THC and CBD (0.1–1 μg/mL) on Caco-2 cell monolayers paracellular permeability in basal condition **(A)**, oxidative stress induced by H_2_O_2_ **(B)** and inflammatory conditions induced by INFγ-TNFα treatment **(C)**. Data are shown as mean ± SEM percentage of basal fluorescent intensity (*n* = 3). *p < 0.05; **p < 0.01 treatment vs. control; §*p* < 0.05 treatment vs. stressor stimulus, unpaired Student’s *t*-test.

Figure 9 shows the effect of THC and CBD treatments on Caco-2 paracellular permeability in basal (A), oxidative (B), and inflammatory (C) conditions. Coherently with TEER measurements, THC is not able to reduce fluorescein isothiocyanate permeability after stimulation (both oxidative and inflammatory). On the other hand, CBD reduced paracellular permeability in oxidative and inflammatory conditions with respect to stimuli, being more effective at 0.1 μg/mL.

### CBD Prevents Epithelial Barrier Damage Maintaining Membrane Integrity

**FIGURE 10:**
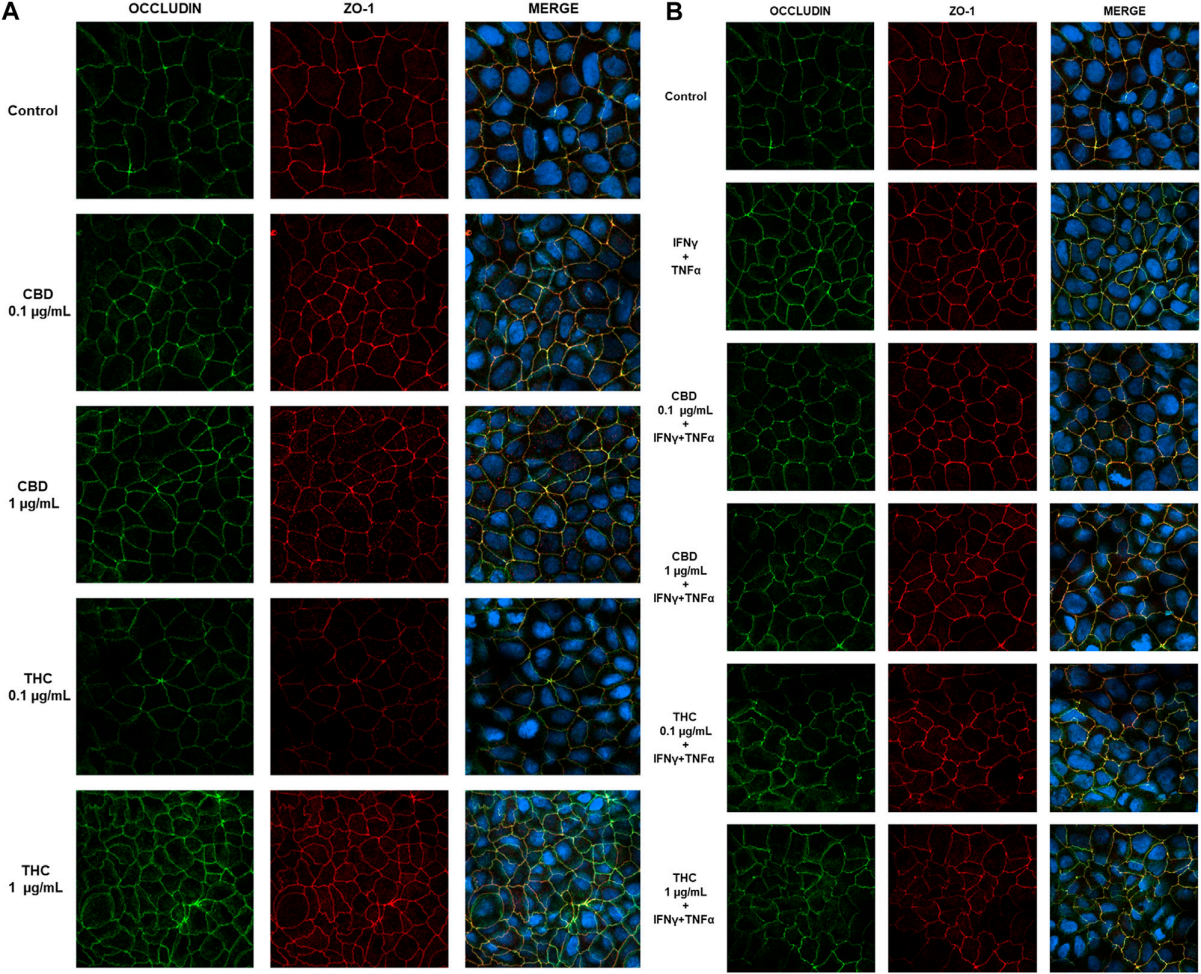
Effect of CBD and THC extracts (0.1 and 1 μg/mL) on occludin and ZO-1 expression in Caco-2 cells. **(A)** Representative images of the effect of CBD-THC treatment on tight junction proteins in Caco-2 cell monolayers. **(B)** Representative images of the effect of CBD and THC on tight junction proteins in inflammatory conditions (INFγ+TNFα stimulation). Images were collected by confocal laser-scanning microscope LSM800 and software ZEN 2.1, magnification 60X and are representative of three experiments.

To confirm the protective effect of CBD in the maintenance of intestinal barrier integrity, the effect on tight junctions was evaluated by confocal microscopy. Occludin and ZO-1 protein location and distribution was evaluated in differentiated Caco-2 cells monolayers stimulated with the inflammatory paradigm (INFγ+TNFα). Images in Figure 10 show that in untreated Caco-2 monolayers, occludin and ZO-1 immunofluorescence signal appears as a continuous belt-like structure encircling cell. CBD maintains the junction’s structure analogous to the control while THC treatment at both the concentrations appear to induce a change in cell morphology, showing tight junction proteins less continuous and more irregular (Figure 10A). Figure 10B shows that treatment with INFγ+TNFα causes alterations in TJ morphology and localization, rendering the membrane ring structure irregular. These alterations in TJ proteins caused by the inflammatory stimuli were prevented by CBD treatment. This data appears to be in line with the one observed in TEER and permeability measurement. CBD is in fact able to prevent the epithelial barrier damage induced by the inflammatory stimulus, preventing membrane disruption.

## Discussion

Inflammatory bowel disease is a set of clinically important chronic inflammatory conditions of the gastrointestinal tract that seriously affect all aspects of patients’ live (Ng et al., 2018). The incidence of IBD is increasing mostly in industrialized countries throughout the world (Burisch et al., 2013), bringing to attention the need of develop innovative effective therapeutic approaches. The current IBD therapy focuses on suppression of the immune system, and current drugs present collateral and side effects that seriously limit their necessary long-term use. As such, complementary and alternative medicine, in particular natural remedy, is becoming popular in IBD treatment and symptoms control. Many natural compounds have been used in clinical trials suggesting the potential use of *aloe vera*, *Boswellia serrata*, tormentil extracts, mastic gum, etc. and some have been proven promising in IBD treatment (Zhao et al., 2017) Herbal therapies exert their therapeutic effect by different mechanisms including maintenance of redox homeostasis, improvement of epithelial barrier integrity, restoration of microbiota homeostasis, immune regulation, etc. (Triantafyllidi et al., 2015). Many intestinal disorders present their etiopathogenesis linked to interactions between altered intestinal permeability and luminal exogenous agents, as well as secretory products of the mucosa itself (Alhamoruni et al., 2012). In recent years, several studies had underlined the involvement of the oxidative stress as well as immune activation as major contributing factors to tissue injury, together with alteration in epithelial permeability that leads to increased and long-lasting exposure of the mucosa to antigens, cytokines, and ROS, inducing a permanent status of inflammation (Moura et al., 2015; Bourgonje et al., 2020).

*Cannabis sativa* has been traditionally used to treat several gastrointestinal disorders and several studies had identified the presence of a functional endocannabinoid system in the gut giving reason to a *Cannabis* effect at GI level (Croci et al., 1998; Kulkarni-Narla and Brown 2000; Coutts et al., 2002; Casu et al., 2003); in particular CB1 receptor is mainly expressed in the gastrointestinal tract of many mammal’s species, including humans. More recent investigations had demonstrated that the endocannabinoid system is strongly activated during inflammations, both in animal models and in tissue samples from patients (Kulkarni-Narla and Brown 2000; Barrie and Manolios 2017; Donvito et al., 2018; Almogi-Hazan and Or 2020). Studies in murine models of colitis (Silvestri et al., 2020) and retrospective observational studies in *Cannabis* users have been done, showing significant improvements of symptoms which translated into less need of medications (Naftali et al., 2013; Storr et al., 2014; Mbachi et al., 2019) but these findings are not necessarily associated with mitigating disease progression or decreasing severity. Clinical trials with *Cannabis sativa* in patients suffering from inflammatory bowel diseases have shown improvement in quality of life but failed to provide evidence for a reduction of inflammation markers (Kienzl, Storr, and Schicho 2020).

The ability to modulate intestinal permeability during inflammation may be an important aspect to consider for therapeutic options to restore a leaky paracellular barrier. Thus, given the traditional benefic effect of cannabinoids in inflammatory intestinal conditions and the observational positive data, in this study we investigated the pharmacological activity of different cannabinoids isolated from *Cannabis sativa* toward the modulation of epithelium function parameters in an *in vitro* model of intestinal inflammation. At this purpose, carcinoma colon cell line (Caco-2) has been used as *in vitro* intestinal epithelial model and the effect of several cannabinoid extracts were tested in inflammatory-mimicking conditions. Beside the others, cannabinol resulted to be the most effective compound isolated from *C. sativa* at our purpose.

Abnormally high levels of ROS are produced in IBD and could be a major contributing factor to tissue injury (Zhu and Li 2012; Bourgonje et al., 2020). The administration of antioxidants with additional anti-inflammatory properties may be beneficial in the treatment of IBD; thus, we tested the potentiality of *Cannabis* extract and its main constituents to modulate ROS levels of cells monolayer. CBD reduces oxidative stress both in basal and in oxidative stress conditions being able to counteract the overproduction of ROS species that are detrimental at epithelial level, inducing intestinal injury. Being the alteration of epithelial permeability a well-known factor involved in IBD development and maintenance, we tested the effect of *Cannabis* compounds on TEER and paracellular permeability of the intestinal monolayer. Interestingly, CBD has been shown to prevent tight junctions alterations in inflammatory conditions, allowing a better maintenance of intestinal epithelial barrier. TEER decrease and paracellular permeability increase, which are hampered by inflammatory stimulus, are prevented by cannabidiol treatment suggesting its protective role in gut homeostasis.

Our study is located in the panorama of research aimed to elucidate the effectiveness of new herbal medicine strategies for IBD. Data acquired in this work underline the role of CBD as a potential modulator of markers of gut inflammation such as ROS production, alterations in the paracellular permeability and transepithelial resistance.

Several studies demonstrate that the non-psychotropic phytocannabinoid CBD may represent the most promising candidate for clinical utilization due to its lack of psychoactive actions (Mechoulam and Hanus 2002). Data collected in this work and in recent years showed that it exerts a wide range of beneficial pharmacological actions on GI functions, ranging from antioxidant to anti-inflammatory and immunomodulatory activities described both in *in vitro* and in acute and chronic animal models of inflammation (Costa et al., 2004, 2007; Nasser, Woo, and Andrews 2020; Skinner et al., 2020) further supporting its potential use in IBD conditions.

## Data Availability

The original contributions presented in the study are included in the article/Supplementary Material, further inquiries can be directed to the corresponding authors.
