# Supplementary material for: Cannabidiol Isolated From Cannabis sativa L. Protects Intestinal Barrier From In Vitro Inflammation and Oxidative Stress
Source: Front Pharmacol. 2021 Apr 28;12:641210. doi: 10.3389/fphar.2021.641210 (PMC8115937; doi:10.3389/fphar.2021.641210)
Supplement: Supplementary file 1 [file image1.pdf]

Supplementary Material

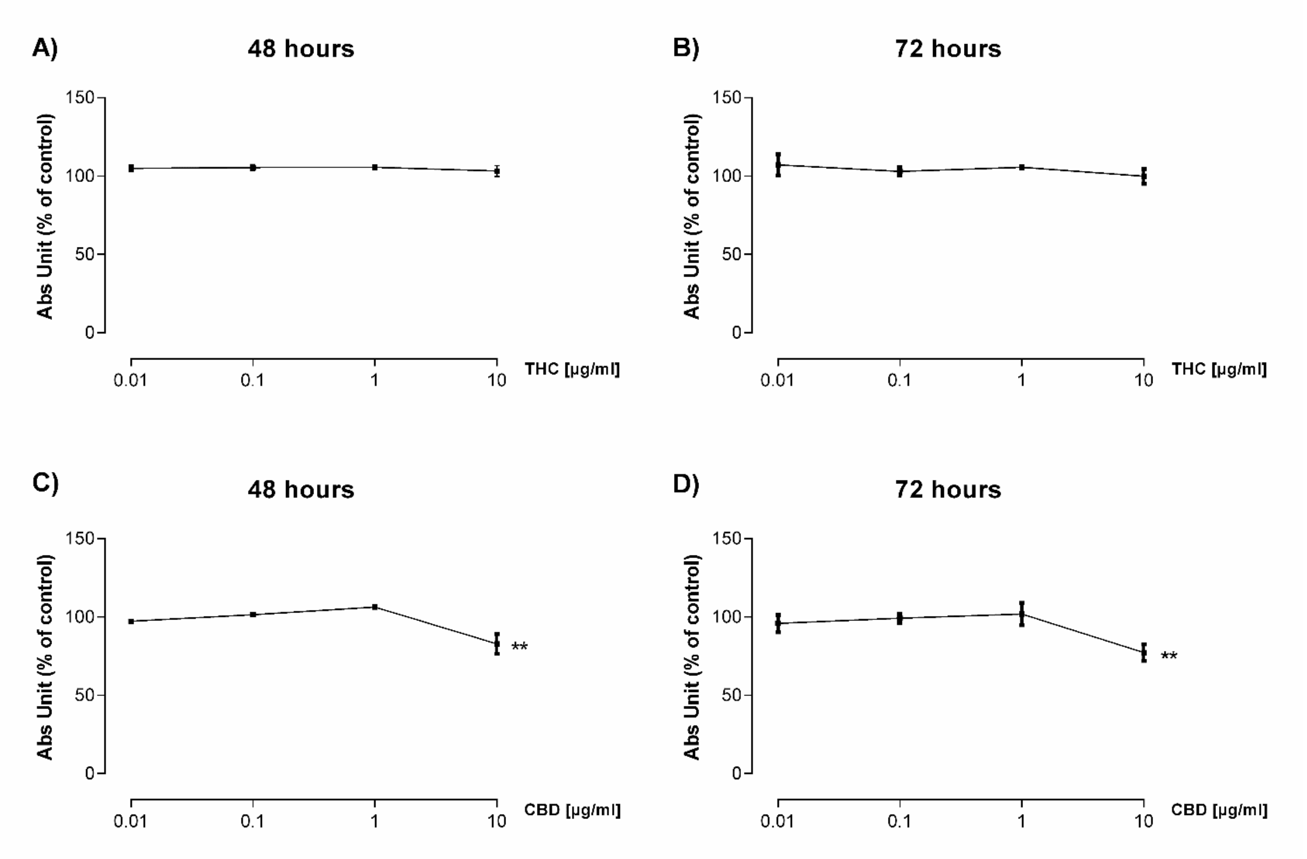

Supplementary Figure 1. Effect of 48-72 h of treatment with THC (A-B) and CBD (C-D) (0.01-0.1-1-10 µg/mL), on Caco-2 cell viability. Results are the mean ± SEM of n = 3 experiments and are expressed as percentage of absorbance of treated cells related to control. **$p<0.01$ treatment *vs* control, Standard ANOVA procedures.
